# To: Long-term psychological outcome after discharge from intensive care

**DOI:** 10.5935/0103-507X.20190014

**Published:** 2019

**Authors:** Fernanda Lima-Setta, Daniela Porto Faus, Franciely Mario Carrijo Campos, Claudia Reis Miliauskas

**Affiliations:** Unidade de Terapia Intensiva Pediátrica, Instituto Fernandes Figueira - Rio de Janeiro (RJ), Brasil. Instituto D'Or de Pesquisa e Ensino - Rio de Janeiro (RJ), Brasil.; Maternidade Escola, Universidade Federal do Rio de Janeiro - Rio de Janeiro (RJ), Brasil. Instituto de Medicina Social, Universidade Estadual do Rio de Janeiro - Rio de Janeiro (RJ), Brasil.; Instituto de Medicina Social, Universidade Estadual do Rio de Janeiro - Rio de Janeiro (RJ), Brasil.; Setor de Saúde Ocupacional, Instituto Nacional de Metrologia, Qualidade e Tecnologia - Rio de Janeiro (RJ), Brasil. Instituto de Medicina Social, Universidade Estadual do Rio de Janeiro - Rio de Janeiro (RJ), Brasil.

To the Editor,

We read with great interest the article "Long-term psychological outcome after discharge from intensive care", by Pereira et al. The authors performed a follow-up study of patients admitted to an intensive care unit (ICU) and evaluated the prevalence of long-term mental health outcomes as well as possible predictors during hospitalization.^(^^1^^)^ The importance of the newly described post-intensive care syndrome is undeniable,^(^^2^^)^ and studies that focus on long-term outcomes that are centered on patients are very relevant.^(^^3^^)^ However, caution is needed when analyzing the results presented.

First, the sample size (n = 17) has low power; thus, one cannot make inferences based on this study. The dissemination of distorted results generated from small samples can affect clinical practice. One should be concerned about liability when disseminating results like these in journals whose audience is composed of professionals who provide care and can make mistaken decisions based on spurious results.

In addition, the internal validity of the study is impaired because of the losses due to follow up. Based on the application of the eligibility criteria, 47 people should have been included in the sample; however, 22 patients were lost before the first measurement, and eight were lost between the first and second measurements, representing a loss of 30 patients (63% of the sample). The characteristics of these 22 patients are not described, and (given this large number) the losses are most likely associated with the outcome and produce biased results.^(^^4^^)^ We suspect the results are biased given the finding that severe hypoxia reduced the chance of developing a long-term cognitive deficit. Most likely, patients with more intense cognitive impairments died or became dependent on the daily routine, thereby comprising the follow-up losses.

Although the secondary objective of the study was to investigate predictive factors, the authors described their results using the term "risk factors". To investigate this type of association, it is necessary to use a causal model that accounts for confounders, mediators, and colliders. This study did not have the necessary research design and could never achieve this objective. The way that the data are presented induces the reader to misinterpret them.

The description of the analysis was confusing, and the use of the various tests and statistical models was inadequate. It was not clear whether the authors performed simple bivariate logistic regressions or a single regression with all of the variables included in the model. In both cases, the excess of tests would favor the appearance of significant associations to appear by chance. Moreover, in the second situation, from the point of view of statistical parsimony, it would not make sense to perform a model using 11 fitted variables with a sample size of only 17 individuals.

We believe that the methods of this research present important problems and produce distorted results, making its discussion inadequate. Severe hypoxia has dramatic consequences for any patient in an ICU,^(^^5^^)^ and the dissemination of biased results might attenuate the attention and care of the healthcare team in such situations and have serious implications for clinical practice.

*Fernanda Lima-Setta* *Pediatric Intensive Care
Unit, Instituto Fernandes Figueira - Rio de Janeiro (RJ),
Brazil* *Instituto D'Or de Pesquisa e Ensino - Rio de
Janeiro (RJ), Brazil* *Daniela Porto
Faus* *Maternidade Escola, Universidade Federal do Rio de
Janeiro - Rio de Janeiro (RJ), Brazil* *Instituto de
Medicina Social, Universidade Estadual do Rio de Janeiro - Rio de Janeiro (RJ),
Brazil* *Franciely Mario Carrijo
Campos* *Instituto de Medicina Social, Universidade
Estadual do Rio de Janeiro - Rio de Janeiro (RJ),
Brazil* *Claudia Reis
Miliauskas* *Occupational Health Sector, Instituto
Nacional de Metrologia, Qualidade e Tecnologia - Rio de Janeiro (RJ),
Brazil* *Instituto de Medicina Social, Universidade
Estadual do Rio de Janeiro - Rio de Janeiro (RJ),
Brazil*
